# Generation of Virus-Free Induced Pluripotent Stem Cell Clones on a Synthetic Matrix via a Single Cell Subcloning in the Naïve State

**DOI:** 10.1371/journal.pone.0038389

**Published:** 2012-06-13

**Authors:** Naoki Nishishita, Masayuki Shikamura, Chiemi Takenaka, Nozomi Takada, Noemi Fusak, Shin Kawamata

**Affiliations:** 1 Riken Center for Developmental Biology, Minatojima-Minamimachi, Chuo-ku, Kobe, Japan; 2 Foundation for Biomedical Research and Innovation, Minatojima-Minamimachi, Chuo-ku, Kobe, Japan; 3 DNAVEC Corporation, Tsukuba, Ibaragi, Japan; 4 Japanese Science and Technology Agency, Kawaguchi, Saitama, Japan; University of Freiburg, Germany

## Abstract

CD34+ cord blood cells can be reprogrammed effectively on dishes coated with a synthetic RGD motif polymer (PronectinF®) using a temperature sensitive Sendai virus vector (SeV TS7) carrying reprogramming factors OCT3/4, SOX2, KLF4 and c-MYC. Dish-shaped human ES cell-like colonies emerged in serum-free primate ES cell medium (supplemented with bFGF) in 20% O2 culture conditions. The copy numbers of SeV TS7 vectors in the cytoplasm were drastically reduced by a temperature shift at 38°C for three days. Then, single cells from colonies were seeded on PronectinF®-coated 96-well plates and cultured under naïve culture conditions (N2B27-based medium supplemented with LIF, forskolin, a MAPK inhibitor, and a GSK inhibitor in 5% O2) for cloning purpose. Dome-shaped mouse ES cell-like colonies from single cells emerged on PronectinF®-coated dishes. These cells were collected and cultured again in primate ES cell medium supplemented with bFGF in 20% O2 and maintained on PronectinF®-coated dishes. Cells were assessed for reprogramming, including the absence of residual SeV and their potential for three germ layer differentiation. Generation of virus-free induced pluripotent stem cell (iPSC) clones from single cells under feeder-free conditions will solve some of the safety concerns related to use of xeno- or allogeneic-material in culture, and contribute to the characterization and the standardization of iPS cells intended for use in a clinical setting.

## Introduction

Induced pluripotent stem cells (iPSCs) can be generated in a chromosome non-integrating manner to reduce the chance of tumorigenicity caused by random chromosomal insertion of exogenous genes. Several non-integrating reprogramming methods using plasmids [1], [2] piggy-back transposons [3], [4], small peptides [5], [6] and protein delivery methods have been reported [7]. Among the vectors employed for these experiments, the Sendai virus (SeV) vector which lacks a DNA phase is recognized as a potent reagent for reprogramming of somatic cells [8]–[10]. However, complete removal of the SeV construct carrying reprogramming factors from transfected colonies is essential to assure three germ layer differentiation of individual cells. The presence of residual reprogramming factors in transfected cells could impede differentiation and contribute to formation of tumors after implantation. Therefore, the possible presence of the SeV genome should be checked at a single cell level (not at a cell clump level) utilizing a single cell cloning technique in the naïve state.

Human ES cells and human iPSCs correspond to mouse “epi-blasts” after implantation with respect to their gene expression profiles and their dish-like morphologies [11]. They can also be passaged as cell clumps. These cells are called epistem or “primed” pluripotent stem cells. They cannot contribute to chimerism when injected into the inner cell mass (ICM) of blastocysts. Murine ES cells are “the ICM-type” or “naïve” ES cells, and are “bona fide” pluripotent stem cells. They are able to contribute to chimerism when injected into the ICM of blastocysts and can be passaged in single cell suspension. Human ES cells or iPSCs can be converted to the “naïve” state by changing the culture conditions [12], [13] (Table S1). Cells cannot be cultured in the naïve state for more than ten passages without forced expression of reprogramming factors such as Oct4 and Klf4. But cells in the naïve state can be maintained robustly for four or five passages, which is enough to conduct single-cell cloning of human iPSC.

Replacement of allogeneic or xenogeneic feeder layer cultures with a feeder-free system is another safety issue that must be addressed in establishing iPSCs. Feeder-free culture systems utilizing laminin 511 [14] or Matrigel [15] have been reported for the maintenance of established iPSCs or ES cells. Further, the generation of iPSCs from fibroblasts on vitronectin-coated dishes and maintenance of iPSCs in chemically defined medium on vitronectin-coated dishes has been reported [16]. However, the generation of iPSCs from suspension cells on substrate-coated dishes has not yet been reported. In addition, human naïve iPSC culturing methods using feeder-free systems have not been documented. These aspects of cultivation are important to ensure the safety of established iPSCs. Here, we report the generation of single cell-derived, virus-free iPSC clones from cord blood cells (CBCs) with temperature-sensitive SeV under feeder-free conditions.

## Results

In currently available iPSCs generation techniques, iPSCs are established only in an adherent form. Therefore, the adhesion of reprogrammed cells to a culture dish is a key initial event for the generation of iPSCs from suspension cells. We hypothesized that coating a culture dish with synthetic peptides that bound to adhesion molecules expressed on suspension cells would facilitate the generation of iPSCs in a feeder-free system. The choice of coating peptides must be determined by the cell type chosen for reprogramming. We used CD34^+^ CBCs to generate iPSC. These cells correspond to hematopoietic stem cells and progenitors having distinct genetic and epigenetic profiles. They carry no post-natal genetic damage from the environment, as they are essentially “day zero” cells. Further, using CBCs as a cell source for iPSCs offers the possibility of collaborating with existing cord blood banks for the procurement of clinical grade HLA-matched CBCs.

**Figure 1:**
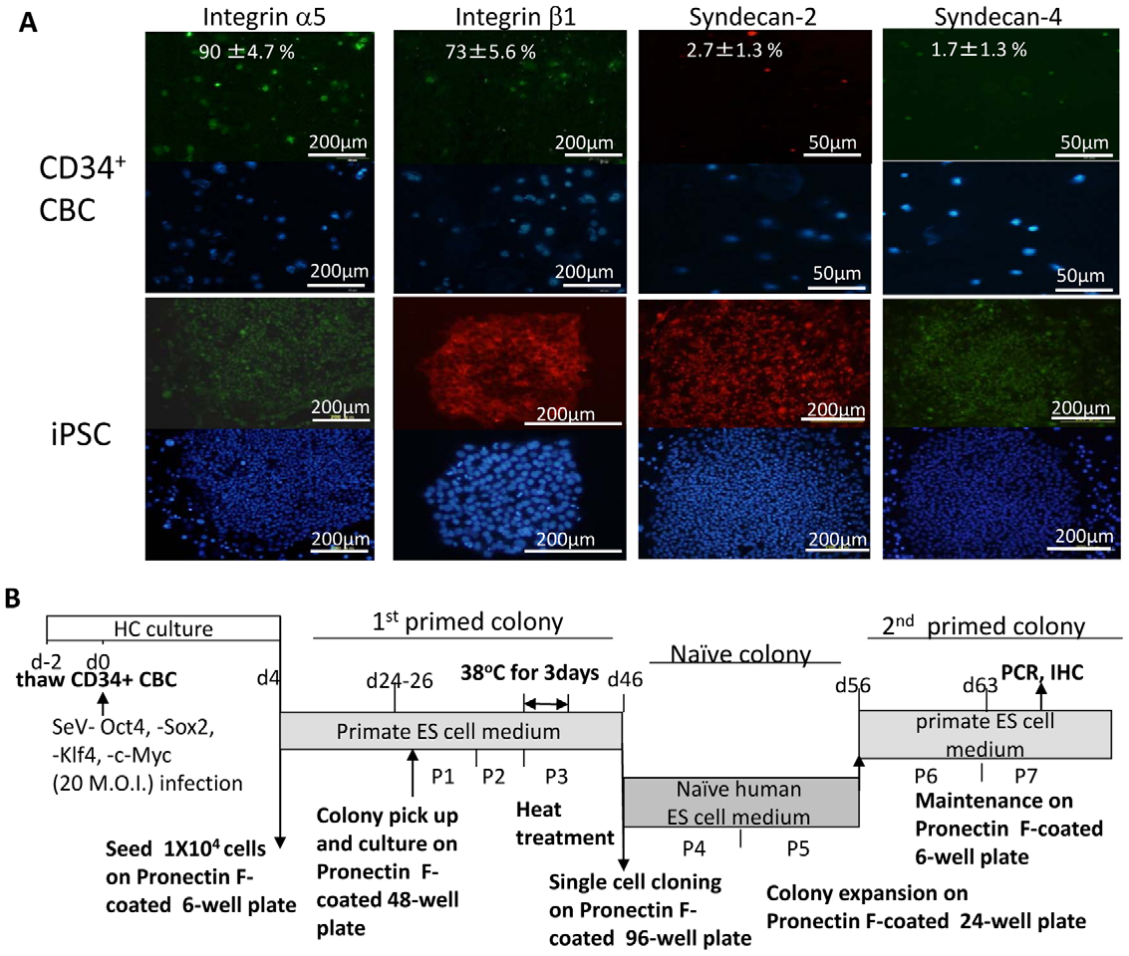
Expression of surface molecules on CD34^+^ cells and iPSCs. (A) Adhesion molecules integrin α5, β1, syndecan-2, and -4 on CD34^+^ CBCs (upper panels) and iPSC colonies (lower panels) detected by immunostaining with the relevant antibody. Alexa 594- and Alexa 488-conjugated secondary antibodies (red and green, respectively) were used to visualize the staining. Nuclei were stained with DAPI (lower photos). Means of the percentages of positive cells with standard deviation are appended in the right top of the photos. (B) Protocol for generation of iPSCs from CD34^+^ CBCs on Pronectin F-coated dishes with temperature sensitive SeV vectors. P: passage.

We used gene chip analysis to determine the levels of adhesion molecule expression on (i) CD34^+^ CBC, (ii) the resulting iPSC on SNL feeder cells [10], (Acc.nos GSM616246), and (iii) naïve iPSC on SNL [17] cultured in naïve cell medium (N2B27-based medium supplemented with LIF, forskolin (a MAPK inhibitor), and a GSK inhibitor in 5% O_2_). We identified several molecules that were expressed by CD34^+^ CBCs and by the resulting primed and naïve iPSCs (Table S1) and the ligands used to anchor suspended CBCs to dishes for reprogramming in a feeder-free system. In agreement with gene chip data, flow cytometric analyses detected integrin α5, and integrin β1, but neither syndecan-2, nor syndecan-4 expression on CD34^+^ CBCs (Figure 1A), while all of these molecules were expressed on iPSCs, as determined by immunochemistry (Figure 1A and Table S4). These data prompted us to use fibronectin, which has an RGD motif that binds with high affinity to the integrin α5/β1 dimer, as a coating material for the generation of iPSCs. Among synthetic peptides having the RGD motif, PronectinF plus® (hereafter referred to as Pronectin F, Sanyo Chemical Industries), which mimics the peptide structure of fibronectin, was chosen and tested. Pronectin F was synthesized by fusing two amino acid motifs, RGD and (GAGAGS)_9_ in tandem to produce a -RGD-(GAGAGS)_9_-RGD-(GAGAGS)_9_-RGD-(GAGAGS)_9_-RGD- polypeptide. This polypeptide (MW 72 kDa) has 13 RGD motifs and is folded at the RGD sequence. Thus, the RGD motif is effectively exposed at the limbs of the peptide bundle, facilitating its potent binding affinity to the integrin α5/β1 dimer.

**Figure 2:**
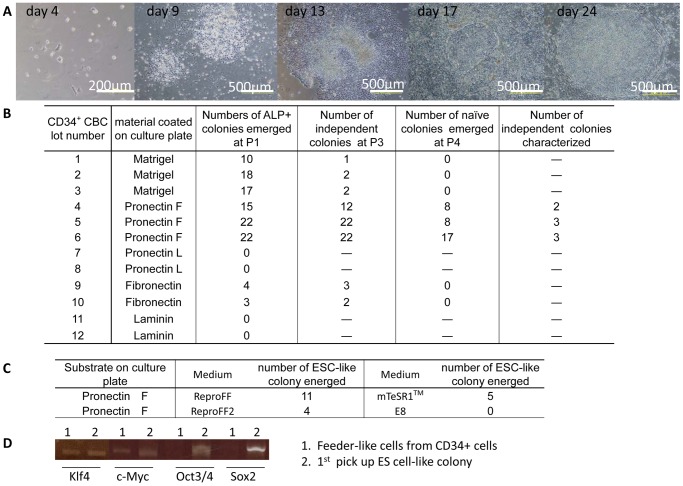
The process of reprogramming and recloning. (A) Phase contrast light microscopic observation of cells during reprogramming and recloning. Images captured on a Pronectin F-coated dish prior to colony picking on days four, nine, 13, 17 and 24 (upper panels). Note that human ES cell-like colonies emerged within a cobblestone like morphology. (B) Efficiency of generating reprogrammed cells on various coating materials and the number of colonies characterized. All the experiments used 1×10^4^ CD34^+^ CBCs, SeV TS vectors at 20 M.O.I. and ReproFF medium. Three independent experiments for Matrigel, Pronectin F and two independent experiments for fibronectin and laminin (Laminin-extracts), Pro*nectin L we*re performed. (C) Frequency of generating human ES cell-like colonies in various culture media. Five thousand CD34^+^ CBCs were infected with 20 M.O.I. of SeV carrying four factors and cultured in ReproFF, ReproFF2, mTeSR1, or E8 medium on Pronectin F-coated dishes to reprogram CD34+ CBCs. D: Endogenous gene expression of *Klf4, c-Myc, Oct3/4,* and *Sox2* in feeder like-cells (lane 1) and first pick up of a human ES cell like-colony (lane 2).

In order to generate iPSCs from CBCs under feeder-free, non-integrating, serum-free conditions, we used a temperature-sensitive SeV vector and serum-free primate ES cell medium. The protocol for generating iPSCs is shown in Figure 1B. The reprogramming process at each passage was monitored by checking the morphology of the reprogramming cells. CD34^+^ cells infected with 20 M.O.I. of SeV constructs were cultured in serum-free hematopoietic cell culture (X-VIVO10, supplemented with SCF, Flt3/4 Ligand, TPO, IL-6 and soluble IL-6 receptor). Some cells attached to Pronectin F-coated dishes by day four (Figure 2A, day four) in agreement with CD34+ cell expression data (Figure 1A). In contrast, no cells attached to Pronectin L®- (hereafter referred to as Pronectin L, Sanyo) 15 days after SeV infection (Figure S1A). Pronectin L is a synthetic peptide having an IKVAV motif and mimicking the protein structure of laminin α1. We were unable to generate any ES cell-like colonies on Pronectin L-coated dishes (Figure 2B). Further, we could not generate ES cell-like colonies on laminin-extract (ReproCELL)-coated dishes (Figure 2B, Figure S1A).

Cobble stone-like cell colonies emerged at day nine and cell clumps with round and small cells emerged inside the colonies at day 13 on Pronectin F-coated dishes (Figure 2A, day 9, day 13). Cell clumps within cobble stone-like colonies grew (Figure 2A, day 17) and finally human ES cell-like colonies emerged (Figure 2A, day 24) on Pronectin F-coated dishes which were then picked up for serial passage. Fifteen to twenty-two dish-shape human ES cell-like colonies were picked out of 10,000 CD34^+^ CBCs seeded on Pronectin F-coated dish in serum-free medium ReproFF (Figure 2B).

Several serum-free media were tested for their ability to support reprogramming of CD34^+^ CBCs on Pronectin F-coated dishes, including ReproFF (ReproCELL), ReproFF2 (ReproCELL), mTeSR1 (STEMCELL Technologies) [18], or the previously reported, chemically defined medium E8 [16]. ReproFF, ReproFF2 and mTeSR1, but not E8 medium supported the adhesion of CD34^+^ CBCs to Pronectin F-coated dishes and the generation of human ES cell-like colonies (Figure 2C). As ReproFF showed superior ability to generate ES cell-like colonies, we used ReproFF for the remaining experiments, unless otherwise specified.

To explore the nature of feeder-like cells that did not generate a human ES cell-like sub-colony within cobble stone-like colonies, the expression of endogenous reprogramming factors such as *Klf4, Oct3/4, Sox2,* and *c-Myc* was examined by RT-PCR 24 days after infection. In contrast to cells from human ES cell-like colonies, the endogenous expression of *Oct3/4* and *Sox2* in feeder-like cells was not observed (Figure 2D), suggesting that reprogramming of feeder-like cells was incomplete. Flow cytometric analysis (Figure S1B, Table S4) showed that these cells possessed mesenchymal cell characteristics.

It has been reported that CBCs are capable of multipotential differentiation [19], and that iPSCs can be generated from CBCs on mouse feeder MEFs with ectopic episomal expression of Sox2 and Oct4 alone [20]. We were unable to generate iPSC from CBCs with SeV control vector alone (without reprogramming factors) or with SeV-Sox2 and -Oct4 alone under feeder-less conditions in ReproFF medium.

**Figure 3:**
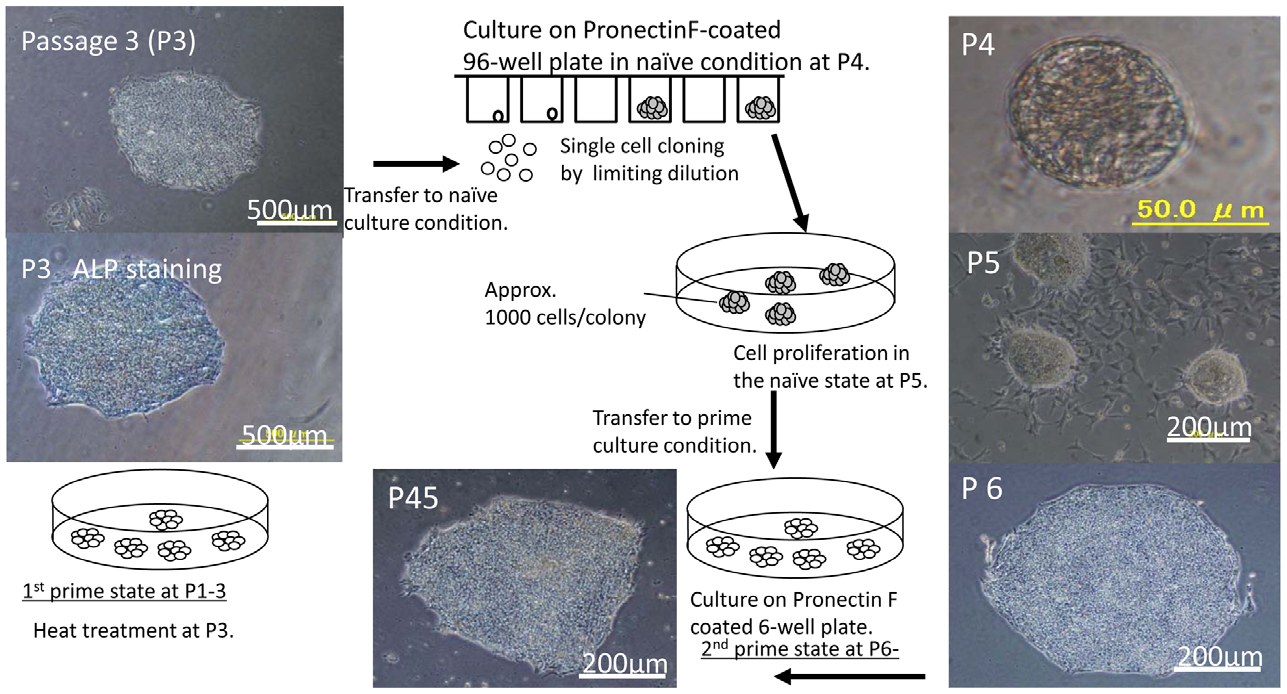
Generation of reprogrammed cell clone from a single cell via the naïve state. Human ES cell-like colonies (first prime state) were picked up at day 24 and cultured on Pronectin F-coated dishes. The colonies were subjected to heat treatment (38°C, three days) at passage three (P3). Light microscopic image and ALP staining at P3 are shown in upper and lower panels, respectively. Colonies emerged from single cells in Pronectin F-coated 96-well plates under naïve conditions at P4, dome-shaped colonies at P5 under naïve conditions, ES cell-like colonies (second primed) cultured under primed culture conditions at P6 or long-term passaged clone (PFX#9) at P45 are shown.

Human ES cell-like colonies in the primed state were selected and named PF (Pronectin F-coated) clones (Table S2A). These clones were maintained for three passages (1^st^ primed, P1–3) and subjected to heat treatment at passage three to reduce the amount of residual SeV after reprogramming (Figure 3, P3). Three hundred single cells from ES cell-like colonies in the primed state were seeded in ten Pronectin F-coated 96 well plates in a limiting dilution manner (approximately one cell per three wells) under naïve culture conditions to generate colonies from individual cells (Figure 3, P4). The frequency of obtaining colonies in the naïve state from single cells harvested from a single primed colony ranged from two to seven colonies per 300 cells seeded (Table S2A). These colonies were named PFXs (Pronectin F-coated X chromosome reactivated) at passage four. Then, cells in the naïve state were seeded in Pronectin F-coated 24 well plates to allow them to proliferate in the naïve state in passage five.

**Figure 4:**
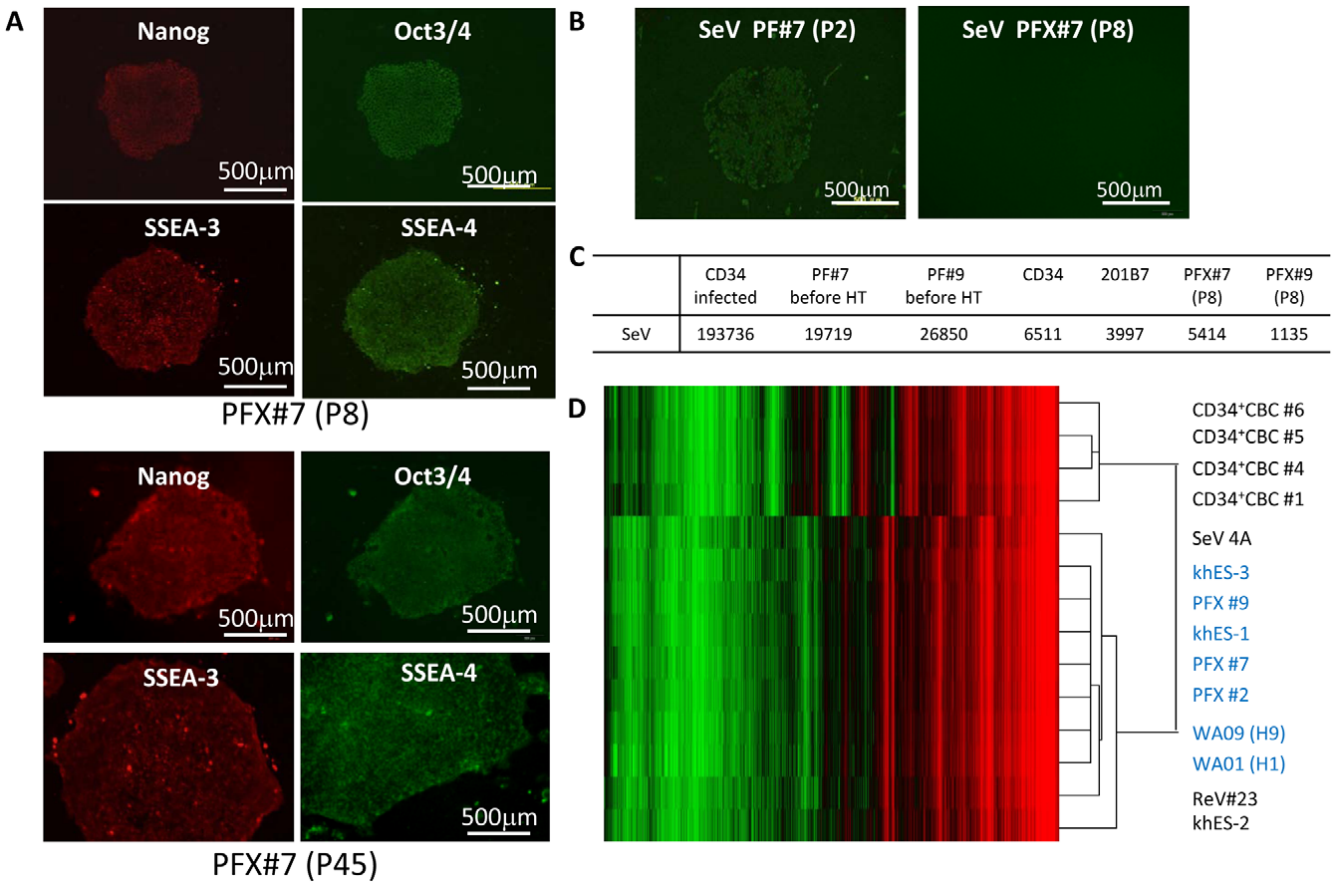
Characterization of single cell-derived reprogrammed clones. (A) Expression of pluripotency-related molecules in reprogrammed cell clones. ES cell-like clone PFX#7 at P8 (upper panels) and at P45 (lower panels) was stained with antibodies against Nanog, Oct3/4, SSEA-3, or SSEA-4 as indicated. Alexa 594- and Alexa 488-conjugated secondary antibodies (red and green, respectively) were used to visualize the staining. (B) Expression of SeV in ES cell-like colonies before heat treatment at passage two (SeV at P2) and after heat treatment and single cell cloning at passage eight (SeV at P8, PFX#7). The SeV construct was determined by immunostaining with antibody against SeV HN. (C) Quantitative RT-PCR determination of residual SeV viral genomes in CD34+ CBCs three days after SeV infection (CD34 infected), first primed colony iPS#7 or iPS#9 before heat treatment at P2 (iPS#7 before HT, TS#9 before HT), non-infected CD34^+^ CBCs (CD34) or iPSC clone generated by retrovirus (201B7), established clones at P9 (PFX#7) or (PFX#9). Values were normalized using the housekeeping gene *GAPDH*. (D) Heat map for gene expression of CD34^+^ CBCs lots #1, #4, #5, #6, established clones PFX#2, #7, #9 at P22, human ES cell lines (khES-1, khES-2, khES-3 at P15 (as obtained), WA01 (H1), and WA09 (H9) at P16 (as obtained) on SNL, iPSC generated from CD34+ CBC by temperature non-sensitive SeV (SeV#4A) at P18 and by retrovirus (ReV#23) at P20 on SNL.

We noted that dome-shaped mouse ES cell-like colonies merged in Pronectin F-coated wells under naïve culture conditions (Figure 3, P5). Gene expression of *Xist* (X-inactive specific transcript) in female cells (XX) in the naïve or in the primed state was assessed by gene chip analysis and suppression of *Xist* gene expression was used as an indicator of being in the naïve state (Table S2B). Harvested cells from passage five were cultured again under primed ES cell culture conditions to maintain stable and long-term passage-able human ES cell like- PFX clones (2^nd^ primed, P6). A total of eight independent PFX clones derived from a single cell were picked up from three different CBC cell sources for further appraisal of colonies in the primed state (Figure 2B). Human ES cell-like PFX clones generated on Pronectin F could be maintained for more than 45 passages without differentiation in the primed state (Figure 3 P45, Figure 4A P45, Figure S3A). Human ES cell-like clones generated and maintained in a feeder-free system could be frozen in cell clumps using DMSO-free, chemically defined and serum-free freezing medium, CryoStem™ Freezing Medium (STEMGENT), and could be cultured again on a Pronectin F-coated dish after thawing. Approximately 10–20% of the colony number scored before cryopreservation in CryoStem™ emerged after thawing. We could generate a passage-able (up to five passages) ES cell-like colony on fibronectin (Sigma)- or Matrigel® (hereafter referred to as Matrigel, BD Life Science)-coated dishes in the primed state, although less effectively compared with that on Pronectin F-coated dishes, but we failed to maintain ES cell-like colonies in the naïve state (Figure 2B).

We assessed the expression of pluripotency-related molecules (Nanog, Oct3/4, SSEA-3 and SSEA-4) by immunostaining at passages eight and 45 (Figure 4A). The remaining SeV constructs were determined by both immunostaining with an anti-SeV HN antibody (Figure 4B) and by quantitative RT-PCR (Figure 4C) prior to and after heat treatment (38°C for three days) followed by single cell cloning. Note that the SeV construct in the established clones after normalization was undetectable or its detection level was equivalent to that of SeV non-infected CD34^+^ CBCs or iPSC 201B7 (Riken BRC) generated by retrovirus [21]. Endogenous expression of pluripotency-related genes was determined by RT-PCR (Figure S2A, Table S3). Karyotypic analyses showed a normal karyotype (Figure S2B). Gene expression profile comparisons with parental CD34^+^ CBC and recloned cell line PFX#7, human ES cell line khES-1 and PFX#7 were also performed (Figure S2C). Flow cytometric analyses of SSEA-4 expression for an established clone are shown in Figure S3C (Table S4). Gene expression comparison studies showed that all PFX clones tested (PFX #2, #7, #9), human ES cells khES-1, khES-3, WA09 (H9) and WA01 (H1) are clustered in the same group. In contrast, iPSC clones we generated from CD34+ CBC by SeV on feeder SNL (SeV 4A) [10] and by retrovirus on SNL (ReV #23) [22] were clustered differently (Figure 4D). These results suggest that PFX clones generated under feeder-less conditions possessed more ES cell-like features (in term of gene expression) than did iPSCs we previously generated on feeders. Further gene expression analysis showed that expression level of some of genes related to Wnt signal and gp130-mediated signal was higher in SeV 4A compared with that in PFX #2, #7, #9, khES-1 and WA09 cluster (Figure. S4). However we are not sure how relevant such difference is to describe the difference in established iPSCs and may need further study to address this issue.

**Figure 5:**
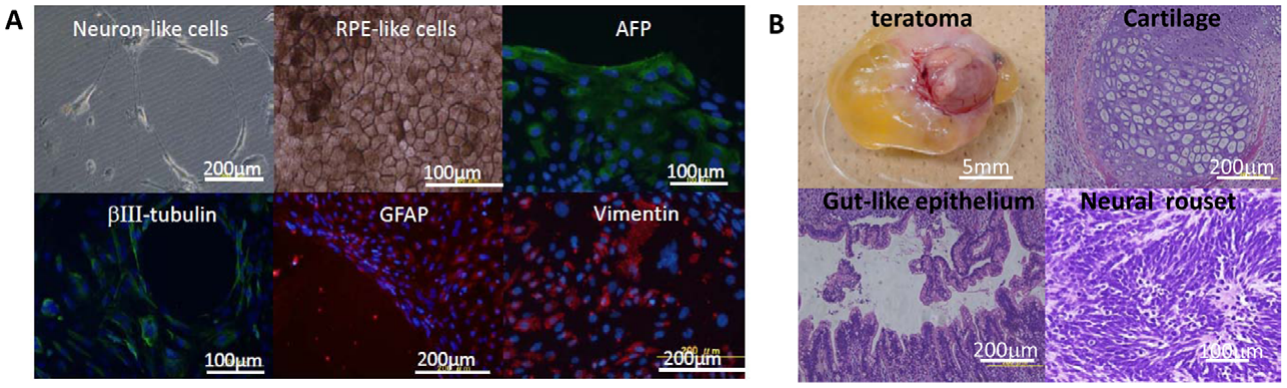
*In vivo* and *in vitro* differentiation potential of established iPSC clones. (A) *In vitro* differentiation potential of established iPSC clones. Phase contrast images of neuron-like (top left) and retinal pigmented epithelium (RPE) differentiation (top middle) of established iPSCs clone PFX#7. Cells were fixed and stained with antibodies against AFP, βIII-tubulin, GFAP and vimentin to identify specific cell lineages. (B) Teratoma with cystic structure derived from iPSCs (PFX #11) implanted in the testicular capsule of NOD-SCID mouse was stained with Hematoxylin and Eosin for histological observation.

The *in vitro* differentiation potential of reprogrammed clones was examined via the EB formation method. The established clones gave rise to cells from all three germ layers, as evidenced by their cell morphology and immunocytochemistry. Namely, we observed cells with neuron-like or retina pigment epithelium (RPE)-like morphology (ectoderm) or cells which were positively immunostained for βIII-tubulin and GFAP (ectoderm), or vimentin (mesoderm), or AFP (endoderm) (Figure 5A, Table S2A, Table S4). Five clones were tested for teratoma formation. Tumors with large cysts were generated some 80 days after transplantation of iPSC clones to the testicular capsules of SCID mice. Hematoxylin and Eosin staining of teratoma tissues showed the derivatives of all three embryonic germ layers, including muscle-like tissue, cartilage (mesoderm), gut-like epithelium (endoderm) and neural rosette-like (ectoderm) tissues (Figure 5B).

## Discussion

Adhesion of suspended cells to the surface of a dish is a key event in reprogramming. Therefore, reprogramming efficiency is critically influenced by the combination of culture dish coating materials, the adhesion molecules expressed by the cells, and the content of the culture medium. In this report, we demonstrated a method for generating iPSC in the absence of a feeder layer as well as a maintenance method. We believe that further studies of synthetic coating materials will lead to the development of an albumin-free, fully defined medium for the reprogramming process. This would eliminate unstable culture conditions arising from the variable quality of albumin. A culture condition which incorporates new synthetic coating materials and albumin-free medium for naïve iPSCs (without forced expression of a set of reprogramming genes) will facilitate effective cell cloning in the naïve state and promote understanding of the “ICM” state of human embryonic development.

Use of a feeder-less culture system to generate iPSCs allowed us to observe the reprogramming process, revealing that the majority of infected cells were incompletely reprogrammed and consequently could not generate ES cell-like colonies. These systems also indicated that only a small portion of infected cells were able to form human ES cell-like colonies. It is uncertain whether incompletely reprogrammed fibroblast-like cells supported the formation of ES cell-like colonies that emerged within fibroblast-like colonies through a feeder-like effect or through a non-autonomous effect (e.g., secretion of growth factors needed for proliferation of reprogrammed cells). However, such effects, if any, might be dispensable for the maintenance of ES cell-like colonies. That is, they could be passaged by cells from human ES cell-like colonies alone, and fibroblast-like cells were not be required nor emerged in the serial passages in the primed culture condition.

The other notable result was that we could not maintain ES cell-like colonies on Matrigel- or fibronectin-coated dishes in the naïve state. The morphology and growth factor requirements of primed and naïve ES cell-like colonies are different. We assume that adhesion molecules and signal pathways required for the maintenance of ES cell-like colonies in the naïve state are distinct from those in the primed state. Moreover, Matrigel containing many growth factors, and fibronectin (MW 440 kDa) having many uncharacterized functional domains may fail to anchor cells effectively and/or promote differentiation of cells under naïve culture conditions. In contrast, Pronectin F, consisting of only functional RGD motifs and lacking a functional domain, could anchor the cells even in the naïve state without triggering differentiation signals in both the prime and the naïve states.

Besides single cell cloning, other possible advantages of culturing iPSCs in the naïve state include standardization of human iPSCs via “decreased” epigenetic diversity (the epigenetic signature) arising from different cell sources [23]. As the naïve state (ICM stage) of human iPSCs is thought to correspond to an earlier developmental stage or less mature stage than the primed state (epi-blasts stage), there is reason to believe that gene expression divergence in the naïve state of human iPSCs decreases compared with that in the primed state. It is intriguing to consider that gene expression divergence of human iPSCs from various cell sources decreases after the naïve (the second primed state) to some extent compared with that in human iPSC established for the first time (the first primed state). Indeed, our preliminary study of gene expression divergence (R^2^) shows that R^2^ values for keratinocyte- and CD34+ cord blood cell-derived iPSCs generated by the same SeV-TS7 vector in the first primed state, naïve state and second primed state were 0.96728, 0.9849 and 0.9761, respectively. The overall divergence in gene expression of two clones from different cell origin in the second primed state decreased after having been in the naïve state. To address this issue, we will have to demonstrate that the differentiation preference or bias reflecting the cell origins found in iPSCs in the first primed state [23] is erased in the second state by a lineage specific*-in vitro* differentiation assay one by one in future studies.

Our defined method for the generation and the maintenance of iPSC by chromosome non-integrated SeV under feeder-free and serum-free conditions will solve some of safety concerns related to tumorigenicity arises from a possible chromosomal integration and infection hazards by using foreign biological products in the culture. And further a single cell cloning of iPSC generated from non-cultured CD34+ CBCs that could have a distinct genetic and epigenetic profile will contribute to the standardization of iPSCs that can be used in a clinical setting in future.

## Material and Methods

All experimental protocols were reviewed and approved by the ethical committee of the Center for Developmental Biology (CDB) Riken, the Foundation for Biomedical Research and Innovation (FBRI), or the animal experiment committee of FBRI.

### Cell Culture

Frozen female CD34^+^ CBCs were supplied by Riken Bio-Resource Center (Riken BRC, Tsukuba, Japan). CD34^+^ CBCs were cultured in hematopoietic culture medium [serum-free X-Vivo10 containing 50 ng/mL IL-6 (Peprotech, London UK), 50 ng/mL sIL-6R (Peprotech), 50 ng/mL SCF (Peprotech), ten ng/mL TPO (Peprotech), and 20 ng/mL Flt3/4 ligand (R&D System, MN)]. Reprogrammed cells were cultured in feeder-less primate ES cell medium Repro FF (ReproCELL, cat. No. RCHEMD004), ReproFF2 (ReproCELL, cat No. RCHEMD006), mTeSR1 (STEMCELL Technologies, catalog number 05850) or E8 (16) supplemented with five ng/mL bFGF (Peprotech) (total bFGF ten ng/mL) on Pronectin F-coated dishes. Passage of human iPSCs was previously described [22]. The split ratio was routinely 1∶3. Human ES cell lines khES-1, -2, -3 were supplied by Riken BRC and WA01(H1), WA09 (H9) were supplied by Wicell (Wisconsin).

### Preparation of Pronectin, Fibronectin or Laminin Coated-dish

One mg/mL stock solution PronectinF plus® (hereafter, Pronectin F, Sanyo Chemical Industries, Kyoto Japan) was prepared by adding one mL of 37°C deionized water to lyophilized Pronectin F. Ten µg/mL of Pronectin F working solution was prepared by diluting the stock solution with phosphate buffered saline (PBS). The culture dish (BD Life Science) was covered completely with Pronectin F and left overnight at room temperature. The coating solution was then removed by aspiration and the dish was rinsed with PBS twice. Coating culture dishes with Pronectin L® (Sanyo Chemical Industries) was performed in the same manner. The culture dish (BD Life Science) was covered completely with fibronectin (Sigma) or laminin extracts (ReproCELL) and left overnight at room temperature. The coating solution was then removed by aspiration and the dish was rinsed with PBS twice.

### Viral Infection and Generation of ES Cell-like Colonies

Temperature-sensitive sendai virus vector (SeV TS) constructs inserting four reprogramming factors (SeV18+OCT3/4/TS7, SeV18+SOX2/TS7, SeV18+KLF4/TS7, SeV(HNL)c-MYC/TS7) were prepared as previously described [10]. Ten thousand CD34^+^ CBCs were transferred to one well of a 96 well plate in 180 µL of hematopoietic cell culture medium. Next, 20 µL of viral supernatant containing 20 M.O.I. each of SeV constructs was added. The medium was changed to fresh medium in the following days (15–18 hours after infection) and infected cells were cultured another three days in hematopoietic culture medium in 96-well plates, after which 1×10^4^ infected CBC were seeded on a Pronectin F-coated six-well dish in human ES cell medium to generate ES cell-like colonies under 20% O_2_ conditions. The medium was changed every day. Colonies were picked at around three weeks after viral infection. Cells from individual colonies were transferred to a Pronectin F-coated 48-well plate to select passage-able ES cell-like colonies. The split ratio was routinely 1∶3. These ES cell-like colonies were further cultured for three days at 38°C to reduce the SeV constructs.

### Single Cell Cloning in the Naïve State

Single cells from dish-shaped (first primed) human ES cell-like colonies at passage three were seeded on a Pronectin F-coated 96 well plate at approximately one cell per three wells and cultured in naïve medium under hypoxic conditions (MCO-5M, SANYO Japan, 5% O_2_, 5% CO_2_ at 37°C). Fifty mL naïve human ES cell medium was prepared by mixing 24 mL DMEM/F12 medium (Invitrogen; 11320), 24 mL Neurobasal medium (Invitrogen; 21103), 0.5 mL×100 nonessential amino acids (Invitrogen), one mL B27 supplement (Invitrogen; 17504044), and 0.5 mL N2 supplement (Invitrogen; 17502048). The medium also contained final concentrations of 0.5 mg/mL BSA Fraction V (Sigma), penicillin-streptomycin (final×1, Invitrogen), one mM glutamine (Invitrogen), 0.1 mM β-mercaptoethanol (Invitrogen), 1.0 µM PD0325901 (Stemgent), 3.0 µM CHIR99021 (Stemgent), ten µM Forskolin (Sigma) and 20 ng/mL of recombinant human LIF (Millipore; LIF1005). After five or six days, dome-shaped mouse ES cell like-colonies were collected and expanded on Pronectin F-coated dishes. Next, cell clumps were transferred to primate ES medium under 20% O_2_ again to culture them in the primed state.

### Quantitative Reverse Transcriptase Polymerase Chain Reaction (qRT-PCR) and RT-PCR

Total RNA was purified with an RNeasy Mini kit (QIAGEN), according to the manufacturer’s instructions. One µg of total RNA was used for reverse transcription reactions with PrimeScript RT reagent kit (TAKARA, Japan). qRT-PCR was performed on am ABI9000 and RT-PCR was performed with an ExTaq (TAKARA, Japan). Primer sequences are shown. See also Table S2.

### Gene Chip Analysis and Karyotyping

Total RNAs from several established iPSCs lines, khES-1 (Riken BRC) and CD34+ CBCs (Riken BRC) were purified with an RNeasy Mini kit (QIAGEN), amplified Ovation Pico WTA System (Takara cat#3300–12), labeled with an Encore Biotin Module (Takara catalog number 4200–12) and then hybridized with a human Gene Chip (U133 plus 2.0 Array Affymetrix) according to the manufacturer’s instructions. Karyotyping of established iPSCs was reported by Nihon Gene Research Laboratories, Inc. (Sendai, Japan).

### Alkaline Phosphatase, Immunohistological Staining

ES cell like-colonies were stained with the Leukocyte Alkaline Phosphatase kit (VECTOR, Burlingame, CA) in accordance with the manufacturer’s instructions. For immunochemical staining, cells were fixed with 4% paraformaldehyde followed by staining with a series of antibodies listed in Table S2. Photomicrographs were taken with a fluorescent microscope (Olympus BX51, IX71, Tokyo) and a light microscope (Olympus CKX31). The percentage of positively stained cells was calculated by scoring the number of antibody-stained and DAPI-stained cells in three independent visual fields.

### *In Vitro* Differentiation

Established ES cell-like clones were transferred to six-well, ultralow attachment plates (Corning) and cultured in DMEM/F12 containing 20% knockout serum replacement (KSR, Invitrogen), two mM L-glutamine, 1% NEAA (Invitrogen), 0.1 mM 2-ME (GIBCO), and 0.5% penicillin and streptomycin to form embryoid bodies (EB). The medium was changed every other day. The resulting EBs were transferred to gelatin-coated plates for 16 days.

### Teratoma Formation

One million iPSCs were injected beneath the testicular capsule of NOD-SCID mice (SLC Japan) for teratoma formation assays. Tumor formation was observed approximately 80 days after cell transplantation. Tumor tissues were fixed with 4% formalin, sectioned, and stained with hematoxylin and eosin.

## Supporting Information

Figure S1 **Characterization of feeder-like cells emerging during the reprogramming process.** (A) Bright field microscopic observation of CBCs on Pronectin L- or laminin (laminin-extracts)-coated dishes 15 days after SeV infection. 1×10^4^ CD34^+^ CBCs were seeded on Pronectin L- or laminin-coated dishes after infection with SeV TS vectors integrated four reprogramming factors at 20 M.O.I. and cultured in ReproFF medium. No adherent cells were observed on Pronectin L-coated dishes (Pronectin L-coated dish). A couple of cell clumps emerged on laminin-coated 24-well plates (Laminin-coated dish, lower panel), but no ES cell-like colony nor feeder-like cells observed. (B) Expression of surface markers as indicated on feeder-like cells six days after infection was determined by flow cytometry.
(TIF)

Figure S2 **Characterization of established iPSC clones.** (A) Expression of endogenous pluripotency related genes in reprogrammed cell clones determined by RT-PCR. (B) Karyotype analysis of established iPSCs clone PFX #7 at passage 20 (P20). (C) Gene expression study comparing parental CD34^+^ CBC #5, human ES cell clone khES01 and the established iPS clone PFX #7. R^2^: dicision coefficient.
(TIF)

Figure S3 (A) Expression of pluripotency-related molecules in reprogrammed cell clones. ES cell-like clone PFX#9 at P8 (upper panels) and at P45 (lower panels) was stained with antibodies against Nanog, Oct3/4, SSEA-3, or SSEA-4 as indicated. Alexa 594- and Alexa 488-conjugated secondary antibodies (red and green, respectively) were used to visualize the staining. (B) Expression of SeV in ES cell-like colonies before heat treatment at passage two (SeV at P2) and after heat treatment and single cell cloning at passage eight (SeV at P8, PFX #9). The SeV construct was determined by immunostaining with antibody against SeV HN. (C) Flow cytometric analysis of established PFX#9 at P8.
(TIF)

Figure S4 **Gene expression comparison study among iPSCs and ESCs.** Gene expression comparison between the mean (mean) expression of five closely clustered pluripotent stem cell lines [two ESCs (H9 and khES-1) and three PFXs (#2, #7 and #9)] and gene expression of ReV#23 (iPSC from CBC with Yamanaka 4factors-Retro Virus on feeder) (A), or that of SeV4A (iPSC from CBC with Yamanaka 4factors-Sendai Virus on feeder) (B)]. C:Gene comparison study of two ESCs (H9 and khES-1) and three PFXs (#2, #7, #9). R^2^: decision coefficient, 6F R^2^: decision coefficient of six pluripotency-related genes (Nanog, Oct4, Sox2, Klf4, Lin28, cMyc). D:List of the genes expressed differently in ReV #23 or SeV4A compared with the mean (± Standard Deviation) of five closely clustered pluripotent stem cell lines (H9, khES-1, PFXs #2, #7 and #9). Yellow cell indicates higher signal value in ReV#23 or SeV 4A and blue cell does lower signal value compared with mean signal value in ESCs/PFXs cluster.
(TIF)

Table S1 **Gene chip analysis of adhesion molecules on CD34^+^ cells, and primed and naive iPSCs cultured on SNL.** Mean and standard deviation of signal values of respective gene expression from three independent experiments is indicated.
(TIF)

Table S2 **(A) Number of colonies established by single cell cloning in the naïve state and a list of tests performed for established clones PFXs.** iPSC clones were generated in Repro FF medium using SeV TS vectors at 20 M.O.I. and Pronectin F-coated dishes. First primed colonies PF #1 - #4 emerging from cord blood cell (CBC) lot #4, PF #5 - #8 from CBC lot #5, PF #9 - #12 from CBC lot #6. (B) *Xist* gene expression analysis by gene chip using four different probes. Naïve PFXs were cultured in the naïve state and 2^nd^ primed PFXs were cultured in the primed state after the naïve state. PF #13 1^st^ prime and khES-1 1^st^ primed were cultured in the primed state (without being in the naïve state). PF #13 and PFXs are female (XX) in origin, while human ES cell line khES01 is male in (XY) origin.
(TIF)

Table S3 **List of primers used for RT-PCR.**
(TIF)

Table S4 **List of antibodies for flow cytometry and immunochemical staining.**
(TIF)
