# Correction: Generation of Virus-Free Induced Pluripotent Stem Cell Clones on a Synthetic Matrix via a Single Cell Subcloning in the Naïve State

**DOI:** 10.1371/annotation/e8fea34f-db11-48e8-b53c-ec7d0733e309

**Published:** 2012-09-18

**Authors:** Naoki Nishishita, Masayuki Shikamura, Chiemi Takenaka, Nozomi Takada, Noemi Fusaki, Shin Kawamata

The fifth author's name was misspelled. The correct name is: Noemi Fusaki. The correct citation is: Nishishita N, Shikamura M, Takenaka C, Takada N, Fusaki N, et al. (2012) Generation of Virus-Free Induced Pluripotent Stem Cell Clones on a Synthetic Matrix via a Single Cell Subcloning in the Naïve State. PLoS ONE 7(6): e38389. doi:10.1371/journal.pone.0038389 

