# Supplementary figures and images for: Generation of Virus-Free Induced Pluripotent Stem Cell Clones on a Synthetic Matrix via a Single Cell Subcloning in the Naïve State
Source: PLoS One. 2012 Jun 13;7(6):e38389. doi: 10.1371/journal.pone.0038389 (PMC3374798; doi:10.1371/journal.pone.0038389)

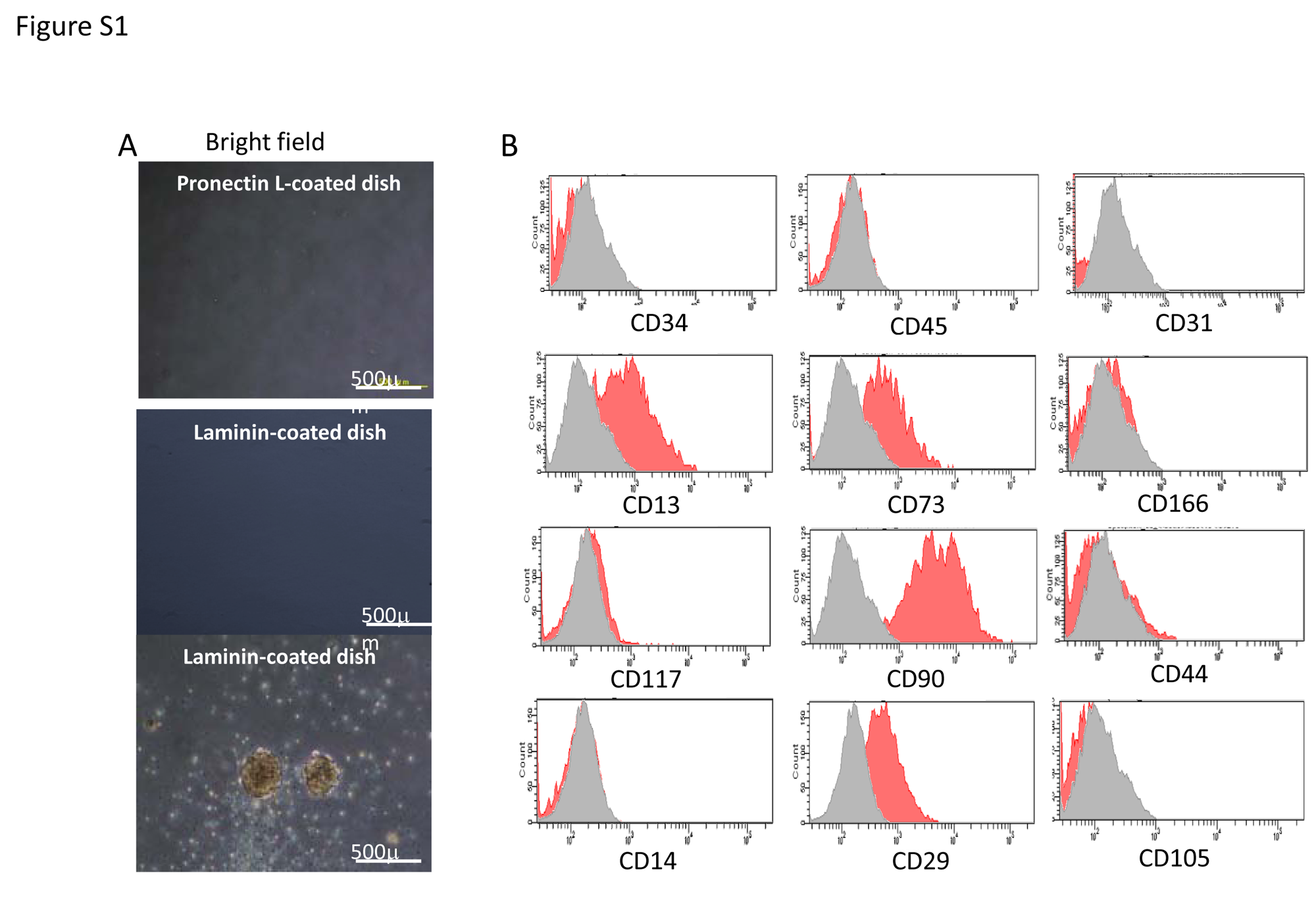

Supplement: Figure S1 — Characterization of feeder-like cells emerging during the reprogramming process. (A) Bright field microscopic observation of CBCs on Pronectin L- or laminin (laminin-extracts)-coated dishes 15 days after SeV infection. 1×10^4^ CD34+ CBCs were seeded on Pronectin L- or laminin-coated dishes after infection with SeV TS vectors integrated four reprogramming factors at 20 M.O.I. and cultured in ReproFF medium. No adherent cells were observed on Pronectin L-coated dishes (Pronectin L-coated dish). A couple of cell clumps emerged on laminin-coated 24-well plates (Laminin-coated dish, lower panel), but no ES cell-like colony nor feeder-like cells observed. (B) Expression of surface markers as indicated on feeder-like cells six days after infection was determined by flow cytometry. (TIF) [file pone.0038389.s001.tif]

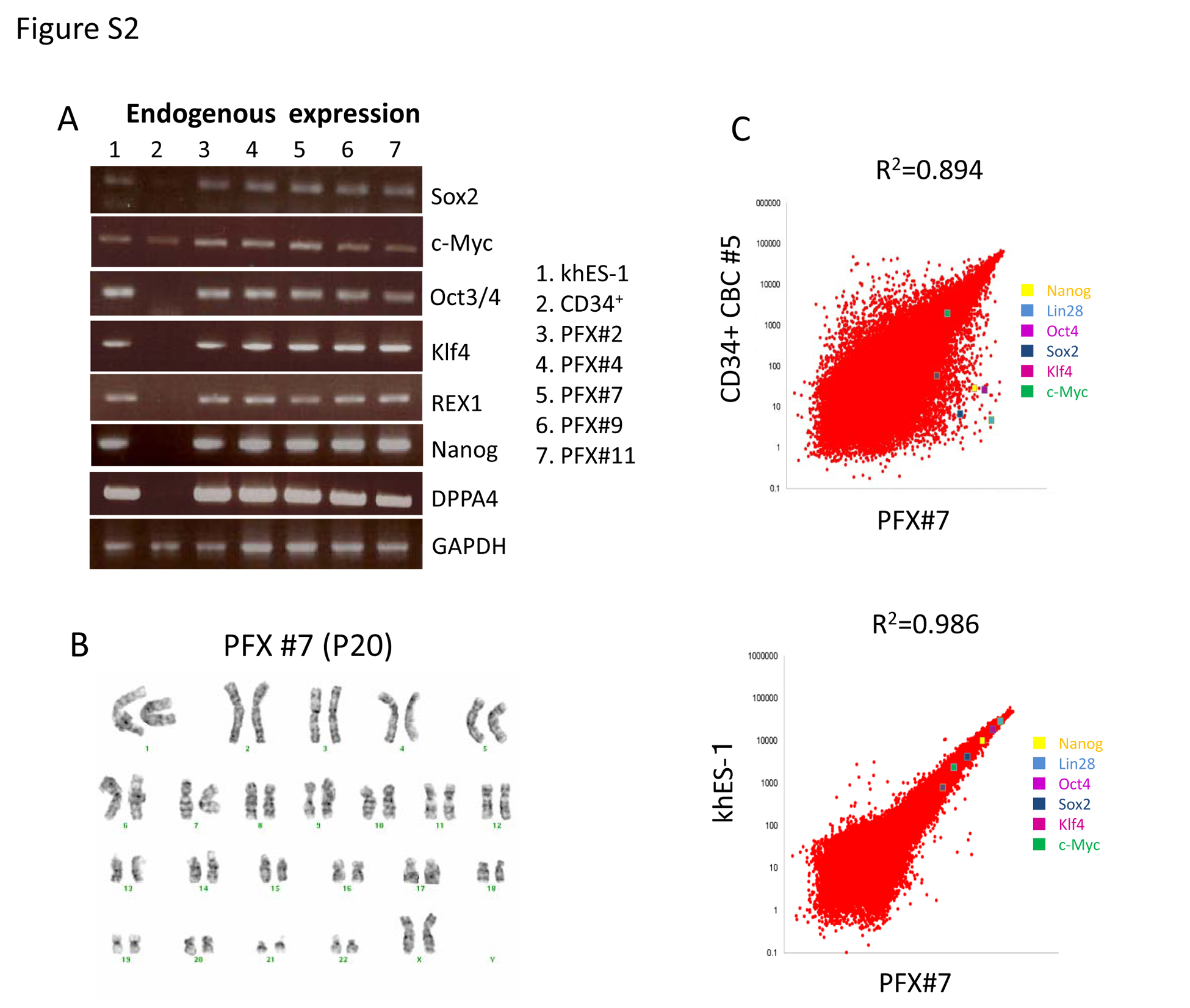

Supplement: Figure S2 — Characterization of established iPSC clones. (A) Expression of endogenous pluripotency related genes in reprogrammed cell clones determined by RT-PCR. (B) Karyotype analysis of established iPSCs clone PFX #7 at passage 20 (P20). (C) Gene expression study comparing parental CD34+ CBC #5, human ES cell clone khES01 and the established iPS clone PFX #7. R2: dicision coefficient. (TIF) [file pone.0038389.s002.tif]

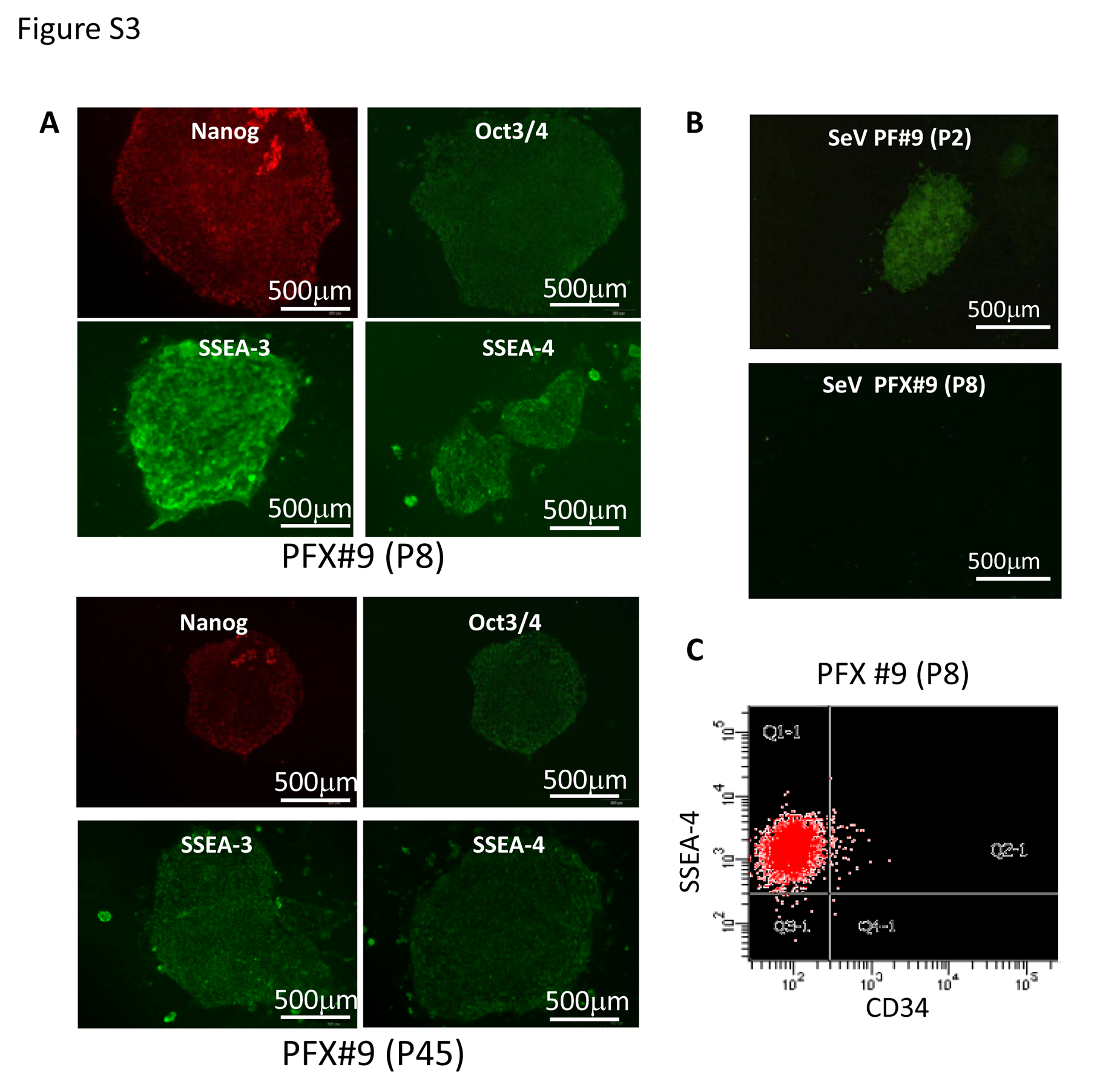

Supplement: Figure S3 — (A) Expression of pluripotency-related molecules in reprogrammed cell clones. ES cell-like clone PFX#9 at P8 (upper panels) and at P45 (lower panels) was stained with antibodies against Nanog, Oct3/4, SSEA-3, or SSEA-4 as indicated. Alexa 594- and Alexa 488-conjugated secondary antibodies (red and green, respectively) were used to visualize the staining. (B) Expression of SeV in ES cell-like colonies before heat treatment at passage two (SeV at P2) and after heat treatment and single cell cloning at passage eight (SeV at P8, PFX #9). The SeV construct was determined by immunostaining with antibody against SeV HN. (C) Flow cytometric analysis of established PFX#9 at P8. (TIF) [file pone.0038389.s003.tif]

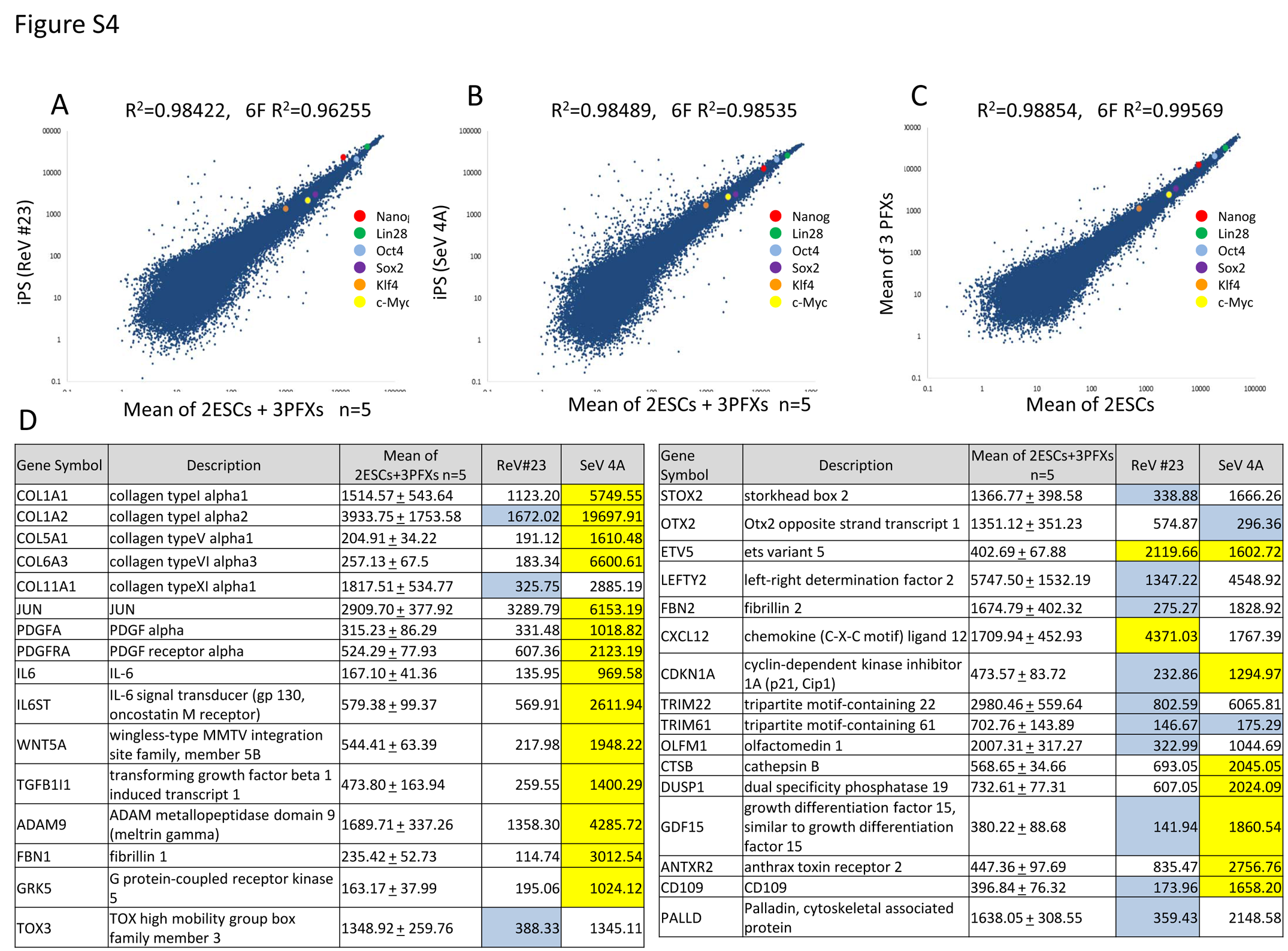

Supplement: Figure S4 — Gene expression comparison study among iPSCs and ESCs. Gene expression comparison between the mean (mean) expression of five closely clustered pluripotent stem cell lines [two ESCs (H9 and khES-1) and three PFXs (#2, #7 and #9)] and gene expression of ReV#23 (iPSC from CBC with Yamanaka 4factors-Retro Virus on feeder) (A), or that of SeV4A (iPSC from CBC with Yamanaka 4factors-Sendai Virus on feeder) (B)]. C:Gene comparison study of two ESCs (H9 and khES-1) and three PFXs (#2, #7, #9). R2: decision coefficient, 6F R2: decision coefficient of six pluripotency-related genes (Nanog, Oct4, Sox2, Klf4, Lin28, cMyc). D:List of the genes expressed differently in ReV #23 or SeV4A compared with the mean (± Standard Deviation) of five closely clustered pluripotent stem cell lines (H9, khES-1, PFXs #2, #7 and #9). Yellow cell indicates higher signal value in ReV#23 or SeV 4A and blue cell does lower signal value compared with mean signal value in ESCs/PFXs cluster. (TIF) [file pone.0038389.s004.tif]

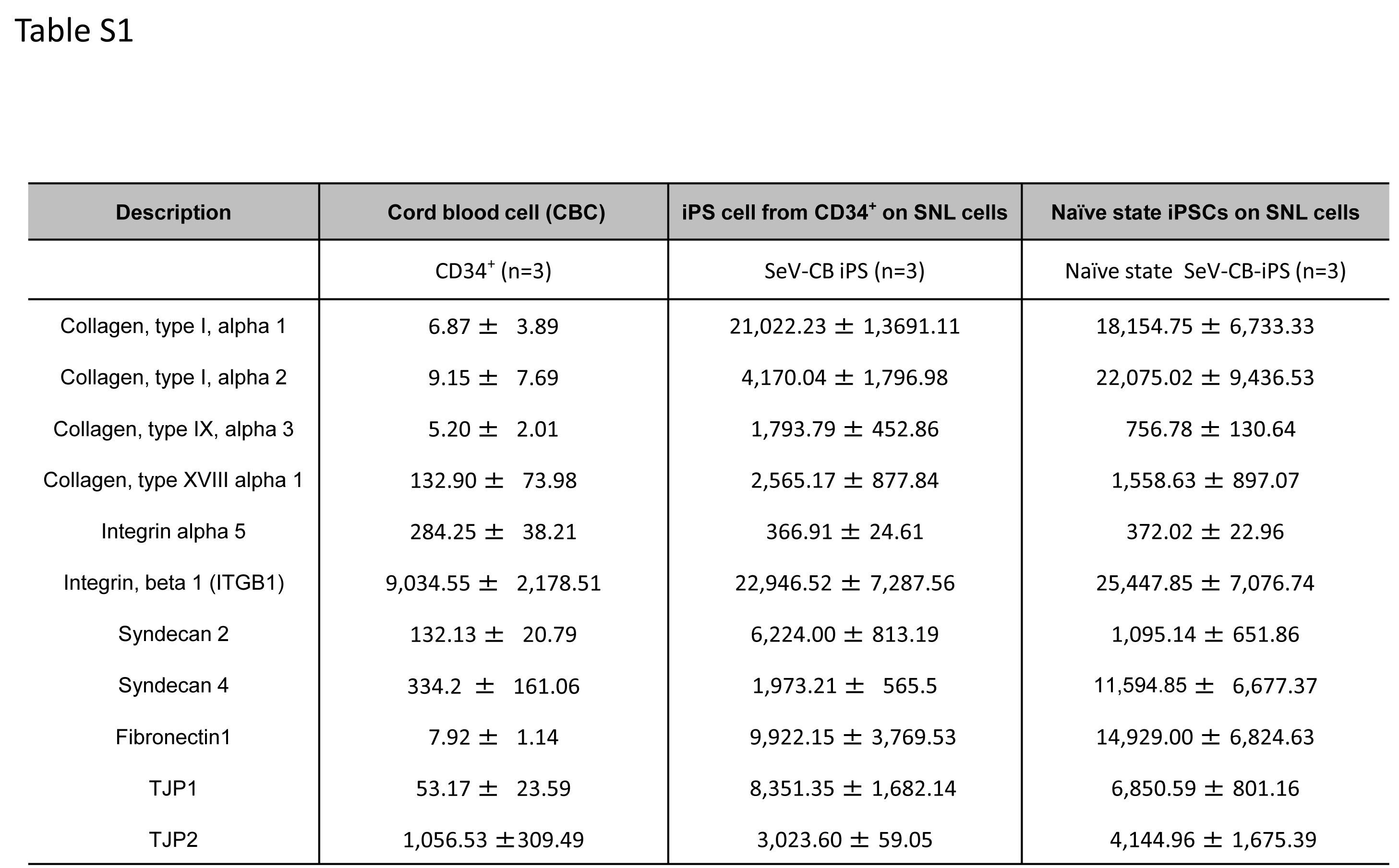

Supplement: Table S1 — Gene chip analysis of adhesion molecules on CD34+ cells, and primed and naive iPSCs cultured on SNL. Mean and standard deviation of signal values of respective gene expression from three independent experiments is indicated. (TIF) [file pone.0038389.s005.tif]

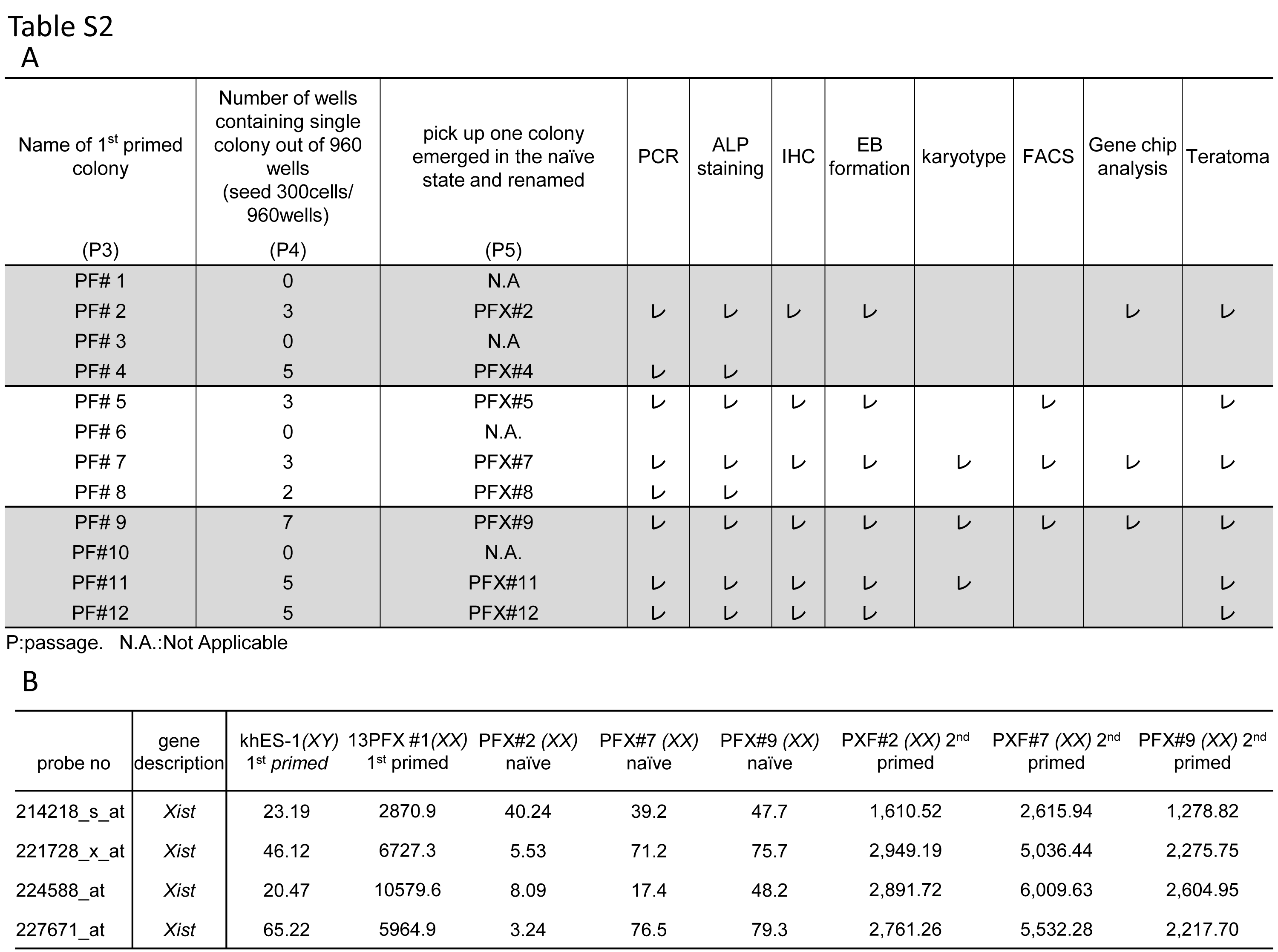

Supplement: Table S2 — (A) Number of colonies established by single cell cloning in the naïve state and a list of tests performed for established clones PFXs. iPSC clones were generated in Repro FF medium using SeV TS vectors at 20 M.O.I. and Pronectin F-coated dishes. First primed colonies PF #1 - #4 emerging from cord blood cell (CBC) lot #4, PF #5 - #8 from CBC lot #5, PF #9 - #12 from CBC lot #6. (B) Xist gene expression analysis by gene chip using four different probes. Naïve PFXs were cultured in the naïve state and 2nd primed PFXs were cultured in the primed state after the naïve state. PF #13 1st prime and khES-1 1st primed were cultured in the primed state (without being in the naïve state). PF #13 and PFXs are female (XX) in origin, while human ES cell line khES01 is male in (XY) origin. (TIF) [file pone.0038389.s006.tif]

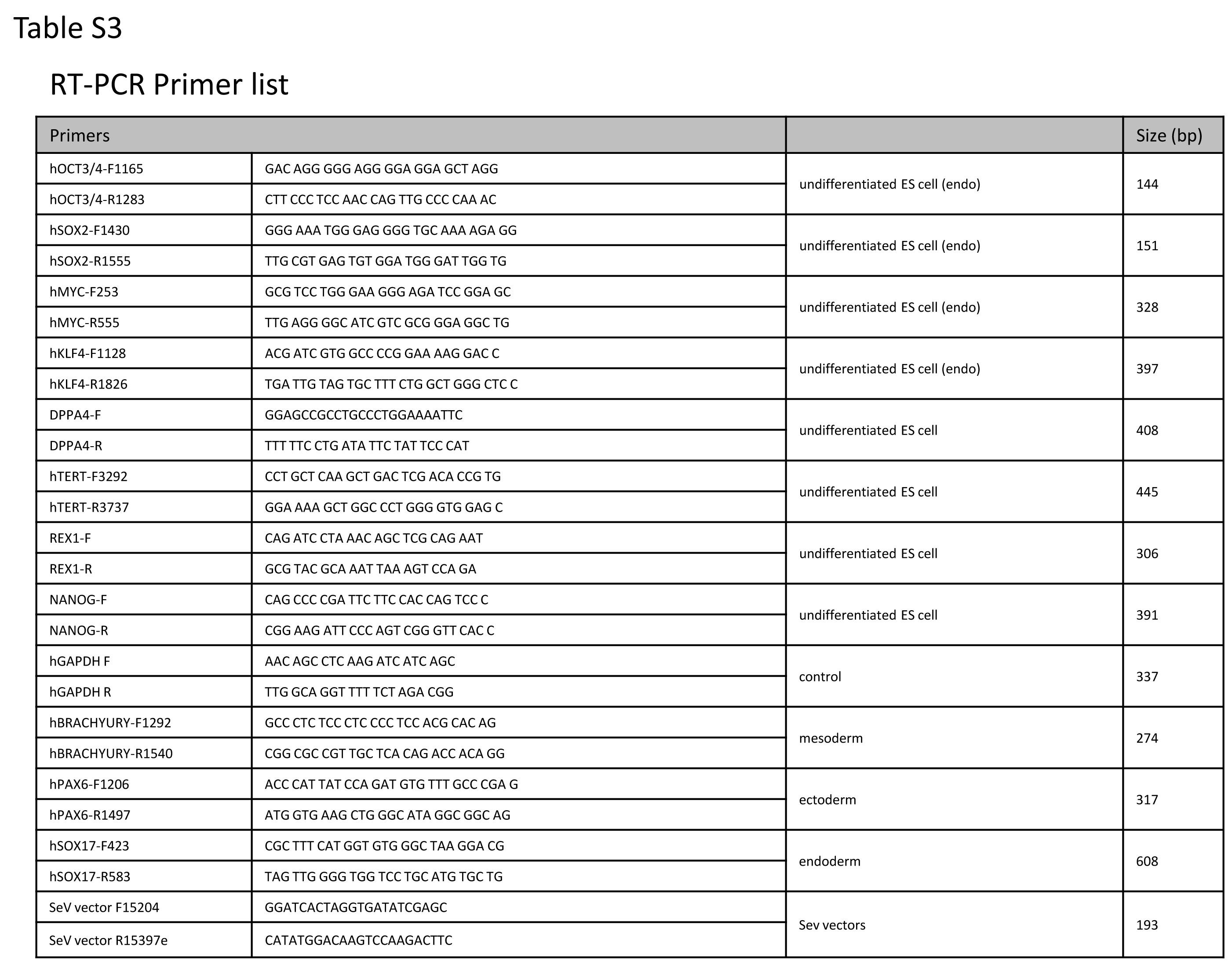

Supplement: Table S3 — List of primers used for RT-PCR. (TIF) [file pone.0038389.s007.tif]

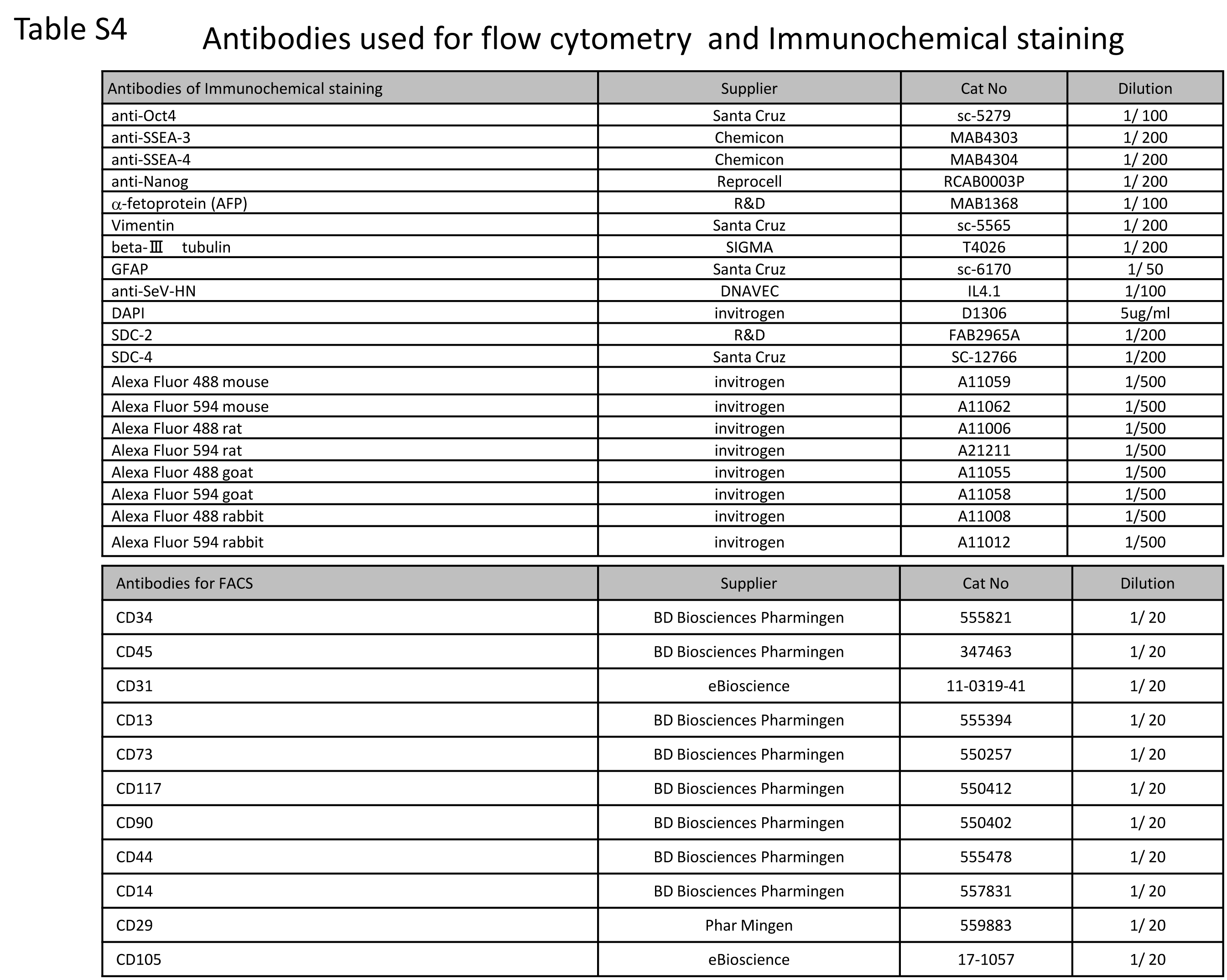

Supplement: Table S4 — List of antibodies for flow cytometry and immunochemical staining. (TIF) [file pone.0038389.s008.tif]
